# FUZ-SMO: A fuzzy slime mould optimizer for mitigating false alarm rates in the classification of underwater datasets using deep convolutional neural networks

**DOI:** 10.1016/j.heliyon.2024.e28681

**Published:** 2024-03-28

**Authors:** Dong liang Zhang, Zhiyong Jiang, Fallah Mohammadzadeh, Seyed Majid Hasani Azhdari, Laith Abualigah, Taher M. Ghazal

**Affiliations:** a School of Computer Science & Technology, Zhoukou Normal University, Zhoukou, 466001, Henan, China; b Engineering Comprehensive Training Center, Guilin University of Aerospace Technology, Guilin, 541004, Guangxi, China; c Department of Electrical Engineering, Imam Khomeini Naval Science University, Nowshahr, Iran; d Hourani Center for Applied Scientific Research, Al-Ahliyya Amman University, Amman, 19328, Jordan; e Department of Electrical and Computer Engineering, Lebanese American University, Byblos, 13-5053, Lebanon; f MEU Research Unit, Middle East University, Amman, 11831, Jordan; g College of Engineering, Yuan Ze University, Taoyuan, Taiwan; h School of Computer Sciences, Universiti Sains Malaysia, Pulau Pinang, 11800, Malaysia; i School of Engineering and Technology, Sunway University Malaysia, Petaling Jaya, 27500, Malaysia; j Center for Cyber Physical Systems, Computer Science Department, Khalifa University, UAE; k Center for Cyber Security, Faculty of Information Science and Technology, Universiti KebangsaanMalaysia (UKM), Bangi, 43600, Malaysia; l Applied Science Research Center, Applied Science Private University, Amman, 11937, Jordan

**Keywords:** Fuzzy slime mould optimizer, LSTM, False alarm rates, Sonar, Dataset classification, Convolutional neural networks

## Abstract

Sonar sound datasets are of significant importance in the domains of underwater surveillance and marine research as they enable experts to discern intricate patterns within the depths of the water. Nevertheless, the task of classifying sonar sound datasets continues to pose significant challenges. In this study, we present a novel approach aimed at enhancing the precision and efficacy of sonar sound dataset classification. The integration of deep long-short-term memory (DLSTM) and convolutional neural networks (CNNs) models is employed in order to capitalize on their respective advantages while also utilizing distinctive feature engineering techniques to achieve the most favorable outcomes. Although DLSTM networks have demonstrated effectiveness in tasks involving sequence classification, attaining their optimal performance necessitates careful adjustment of hyperparameters. While traditional methods such as grid and random search are effective, they frequently encounter challenges related to computational inefficiencies. This study aims to investigate the unexplored capabilities of the fuzzy slime mould optimizer (FUZ-SMO) in the context of LSTM hyperparameter tuning, with the objective of addressing the existing research gap in this area. Drawing inspiration from the adaptive behavior exhibited by slime moulds, the FUZ-SMO proposes a novel approach to optimization. The amalgamated model, which combines CNN, LSTM, fuzzy, and SMO, exhibits a notable improvement in classification accuracy, outperforming conventional LSTM architectures by a margin of 2.142%. This model not only demonstrates accelerated convergence milestones but also displays significant resilience against overfitting tendencies.

## 1 Introduction

The underwater domain represents a mysterious and expansive frontier distinguished by its immense size [1], lack of visibility [2], and intricate nature [3]. Sonar sound datasets play a crucial role in various domains, including underwater surveillance [4], marine research [5], environmental monitoring [6], and naval operations within the vast aquatic environment [7]. The datasets produced by sonar systems possess a distinct capability to investigate the various depths of oceans, lakes, and rivers, thereby revealing concealed structures, topography, and submerged objects [8]. Sonar systems are capable of emitting sound waves and subsequently capturing the resulting echoes, thereby offering significant insights into underwater environments and the objects present within them [9].

The analysis and categorization of sonar sound datasets play a crucial role in effectively utilizing their capabilities, thereby attracting significant attention from researchers in the field. The attainment of precise and dependable categorization holds the utmost importance in various endeavors, including the discernment of submerged entities, marine life, and geological characteristics [10]. Nonetheless, the classification task is confronted with considerable challenges due to the intricate nature of underwater acoustics [11], the diverse characteristics of sonar signals, and the existence of various sources of noise and interference [12].

In light of these challenges, researchers have resorted to employing sophisticated machine-learning methodologies, specifically CNNs and LSTM networks [13]. CNNs have demonstrated exceptional proficiency in extracting intricate features from images [14,15]. On the other hand, LSTM networks exhibit remarkable capabilities in processing sequential data, rendering them well-suited for the analysis of time-series data, such as sonar sound datasets [16]. Nevertheless, achieving the best possible performance of LSTM networks relies on careful hyperparameter tuning, a task that frequently requires significant computational resources [17,18].

Historically, the optimization of hyperparameters has been addressed using techniques such as grid search and random search [19]. Although these methods are efficient, their computational requirements can be high, which can impede advancements in attaining optimal model performance [20]. This study investigates the incorporation of the FUZ-SMO into LSTM hyperparameter tuning in order to improve the classification accuracy of sonar sound datasets, acknowledging the necessity for novel methodologies.

The FUZ-SMO algorithm is derived from the adaptive behavior exhibited by slime moulds, and it introduces a unique approach to optimization [21]. This research aims to improve sonar sound dataset classification by fusing the flexibility of FUZ-SMO with the efficiency of DLSTM's sequence data processing skills. Our study aims to combine the strengths of both domains, resulting in improved classification outcomes, accelerated convergence, and increased resilience against overfitting.

This paper presents a framework that integrates CNNs, LSTM networks, and FUZ-SMO for underwater data classification. The primary objective of this framework is to mitigate false alarm rates effectively. In this study, we present empirical evidence to support the superior performance of the amalgamated model compared to conventional LSTM architectures. This finding signifies a significant breakthrough in the domain of sonar sound classification. The efficacy of this methodology presents encouraging possibilities for the identification of submerged entities, exploration in marine sciences, and diverse applications within the underwater realm, thereby equipping the scientific community with a sophisticated and effective instrument for furthering our comprehension of underwater ecosystems.

To sum up, the primary objective of this study is to accurately classify sonar sound datasets, which is made difficult by the complex characteristics of underwater acoustics and the variability of sonar signals. The objective of our experiment is to evaluate the efficacy of combining the FUZ-SMO with CNNs and LSTM networks. The objective of this integration is to enhance the precision of categorization and minimize the occurrence of false alarms in the classification of undersea datasets. This integration will effectively address a notable deficiency in existing approaches. The tests aim to confirm the notion that the combination of FUZ-SMO, CNNs, and LSTMs can achieve better performance than standard models in this intricate classification problem.

The decision to utilize FUZ-SMO is based on its innovative methodology for effectively managing the trade-off between exploration and exploitation during the optimization process. FUZ-SMO, a novel way to explore the hyperparameter space, combines the principles of fuzzy logic with the adaptive characteristics of slime moulds. Unlike standard approaches like grid and random search, FUZ-SMO offers a dynamic and efficient solution. Complex models such as CNNs and LSTMs can significantly benefit from this approach since it helps mitigate the high computational costs and inefficiencies typically associated with older approaches. The FUZ-SMO approach is presented to optimize the hyperparameter tuning process, resulting in improved and efficient model performance, mainly when working with sonar sound datasets.

The incorporation of CNNs and LSTM models in our proposed framework is motivated by their mutually beneficial capabilities. CNNs are very suitable for processing sonar sound spectrograms due to their expertise in extracting intricate characteristics from data resembling images. Nevertheless, they do not possess the ability to handle sequential dependencies that are inherent in time-series data efficiently. LSTMs are utilized to capture long-term dependencies and patterns in sequence data effectively. Our system intends to enhance the classification of sonar sound datasets by leveraging the feature extraction skills of CNNs and the sequential data processing strengths of LSTMs. This technique is designed to be more thorough and accurate. The proposed integration of these models is believed to overcome the inherent constraints of using each model separately, resulting in a robust solution for the issues encountered in classifying undersea datasets.

The structure of the paper is as follows: Section 2 of this paper provides an overview of the relevant literature, encompassing a comprehensive review of existing scholarly works, an analysis of the advantages and disadvantages of previous approaches, and the identification of the research void that this study aims to address. Section 3 presents the relevant terminology, encompassing the LSTM, CNN, and SMO algorithms. Section 4 provides an exposition of the proposed methodology, encompassing a comprehensive outline of the proposed framework, the amalgamation of CNNs, LSTMs, and FUZ-SMO, a comprehensive overview of the dataset as well as the strategy employed for hyperparameter tuning. Section 5 provides experimental design and evaluation metrics utilized in the study. Section 6 provides an exposition of the results and subsequent analysis, which encompasses a comparative evaluation of the baseline methods as well as a comprehensive discussion of the experimental outcomes. In conclusion, Section 6 serves as the final section of the research, providing an overview of the results and proposing potential avenues for future research.

## 2 Literature review

The categorization of underwater imagery and sonar sound datasets is significant in diverse fields, such as marine research and underwater surveillance [[22], [23], [24]]. This section offers a thorough examination of pertinent literature, focusing on the merits and drawbacks of current methodologies while underscoring the research void that serves as the impetus for our proposed framework.

Numerous research investigations have employed deep learning methodologies for the purpose of classifying underwater images and sonar sounds [[25], [26], [27]]. For example, Madhan et al. [28] utilized deep learning techniques to identify and categorize submerged entities, yielding encouraging outcomes. Nevertheless, one limitation of these methodologies is their dependence on manually adjusted hyperparameters, which makes them computationally demanding and less appropriate for real-time implementations.

The utilization of transfer learning has been investigated as a means to adapt pre-existing models for underwater image classification [29]. Although the approach mentioned above has exhibited enhanced efficacy, it does not explicitly tackle the intricacies associated with sonar sound datasets or the optimization of hyperparameters, thereby creating an opportunity for additional enhancement.

The optimization of LSTM networks in sequence classification tasks heavily relies on hyperparameter tuning [30]. While this study offers valuable insights into optimization techniques, it does not explicitly focus on the classification of underwater images and sonar sounds.

Reference 8 provides an overview of the difficulties encountered in the classification of sonar sound datasets, offering insights into the complexities inherent in this particular field of study. However, this study does not put forth specific strategies to tackle these challenges effectively.

Several studies have employed a combination of various deep-learning architectures for the purpose of classifying underwater images [31]. Although these models exhibit competitive performance, they lack the incorporation of optimization algorithms and fail to address the classification of sonar sound datasets.

In reference proposed by Chen et al. [32], a comprehensive survey is provided on a range of algorithms inspired by the slime mould, with a particular emphasis on their potential applications in the field of optimization. Nevertheless, the paper fails to explore the precise integration of these algorithms with LSTM networks in the underwater domain.

The literature review highlights a notable research deficiency: the lack of a comprehensive methodology that effectively tackles the distinct obstacles associated with classifying sonar sound datasets [33], utilizes deep learning methodologies [34], and efficiently optimizes hyperparameters [35]. Current methodologies frequently depend on manual adjustment or fail to investigate the incorporation of bio-inspired optimization algorithms, such as the SMO.

Recently, a number of academics have attempted to address NP-hard issues in the field of data processing by utilizing the power of stochastic-based techniques, such as evolutionary [36] and heuristics, including whale optimization algorithm [37], simulated annealing [38], grey wolf optimizer [9], dragonfly algorithm [23], biogeography-based optimization algorithm [39], gravitational search algorithm [40], chimp optimization algorithm [41], particle swarm optimization [42] etc. Conversely, the No Free Lunch (NFL) theory [43] shows that no optimization technique can guarantee the optimal solution to all optimization problems and that testing different algorithms is the only way to determine which one works best. As an outcome, this research topic is still drawing researchers. On opposing grounds, Li et al. provide proof that SMO [21] might serve as a possible meta-heuristic technique that prevents trapping in local minima associated with NP-hard situations. These considerations prompted us to utilize SMO for the improvement of CNN's parameters to create a reliable and successful underwater false alarm detection system.

As with other meta-heuristic techniques, SMO struggles when applied to challenges in engineering requiring a highly dimensional search area [44] due to its temporal complexity, poor convergence rate, and propensity to become stuck in local minima. Both of these approaches also share the division of the search space into investigation and utilization phases [45]. First, the population displays erratic behavior as the method searches for a suitable region of probable solutions. However, the most trustworthy solution is reached during the exploitation stage. However, due to the random nature of meta-heuristic techniques, there is no hard line between the two stages. In order to rephrase, when these two periods are not equal, algorithms get stuck at a local minimum. Therefore, in the following, we employ Fuzzy maps (hereafter referred to as FUZ-SMO) to ease a shift across these two stages.

The primary objective of our proposed framework is to address the existing research gap by integrating CNNs, LSTMs, and FUZ-SMO. The proposed methodology aims to enhance both the precision of classification and the optimization of hyperparameters, resulting in improved computational efficiency and resilience. The significance of this approach resides in its capacity to transform the discipline, providing a sophisticated tool for the identification of submerged entities and propelling the progress of scientific investigations in marine environments.

## 3 Relevant terminology

This section presents the relevant terminology encompassing the LSTM, CNN, and SMO algorithms.

### 3.1 Deep long short-term memory

**Fig. 1:**
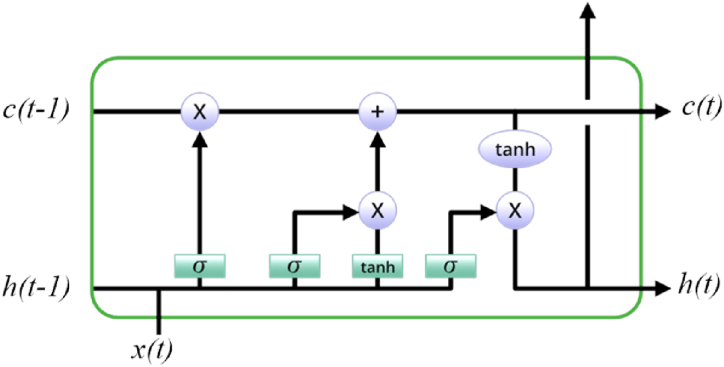
The LSTM in its most common form.

In order to comprehend and forecast sequences, numerous researchers have employed the utilization of the DLSTM structure within recurrent neural networks (RNNs). LSTM networks are constructed using memory cells that possess the ability to retain data over extended durations. This feature allows the network to successfully retain and make use of crucial data from earlier time steps. LSTM models are equipped with gating mechanisms that regulate the information flow within the network. These gating mechanisms consist of the input gate, forget gate, and output gate. In order for the LSTM model to accurately prioritize which pieces of information to keep and which to throw away, the gates mentioned above play a critical function in regulating the data flow into and out of the memory cells. A typical representation of LSTM is shown in Fig. 1 [46].

**Fig. 2:**
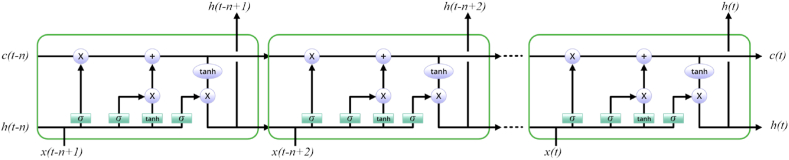
The representation of a typical DLSTM.

Utilizing a hierarchical arrangement of several LSTM layers is what is meant by “DLSTM networks.” The neural network's layers possess the capacity to capture diverse levels of abstraction, thereby facilitating the acquisition of complex patterns throughout the inputs. The LSTM passes the output of one layer to the next in order to create a hierarchical description of the data being fed through a neural network algorithm [47]. A typical illustration of a DLSTM is shown in Fig. 2.

The initial step in the DLSTM process entails the activation of the forget gate, represented as fg(t). This gate holds significant importance as it determines the information that needs to be eliminated from the prior state by the memory cell. Eq. (1) is a mathematical expression for the forget gate, which is symbolized by the fg(t) [46]:

(1) $$fg\left(t\right)=\sigma \left(\alpha_{fg}x\left(t\right)+\beta_{fg}h\left(t-1\right)+\delta_{fg}\right)$$

We use the logistic sigmoid equation (σ). The bias vector can be labeled as $\alpha_{fg}$, $\beta_{fg}$, and $\delta_{fg}$, and the matrix of weights can be changed as needed.

The next step, which identifies the data that is required to be included in the memory cell block to facilitate the update process, follows. The sigmoid coefficient determines the input gate, denoted by *i*(*t*), and uses it to determine which values should be modified. Furthermore, the utilization of a tangent hyperbolic (*tanh*) layer is implemented to generate a potential update vector, which is denoted as *C*(*t*). Eqs. (2), (3) present a comprehensive elucidation of the computational procedure for determining the values of *i*(*t*) and *C*(*t*) [46].

(2) $$i\left(t\right)=\sigma \left(\beta_{i}h\left(t-1\right)+\alpha_{i}x\left(t\right)+\delta_{i}\right)$$

(3) $$c\left(t\right)=\tanh\left(\beta_{c}h\left(t-1\right)+\alpha_{c}x\left(t\right)+\delta_{c}\right)$$

The equations involve a vector that takes on values within the range of 0–1. After deciding which information to keep and discard, the update procedure can proceed by computing the cell state, denoted by *C*(*t*), using Eq. (4) [46]:

(4) c(t)=i(t)∘c(t)+fg(t)∘c(t−1)

The symbol “∘” utilized in this context represents the concept of element-wise multiplicity. The term “fg(t)∘c(t−1)” is employed to denote accumulated data that may potentially be overlooked, while the term “∘c(t)” is utilized to symbolize novel data that will be incorporated into the cell state. The procedure, as shown by Eqs. (5), (6), involves multiplying the result of the hyperbolic tangent by the function *o*(*t*) [46]:

(5) $$o\left(t\right)=\sigma \left(\alpha_{o}x\left(t\right)+\beta_{o}h\left(t-1\right)+\delta_{o}\right)$$

(6) h(t)=o(t)∘tanh(c(t))

Additionally, the parameters $\alpha_{o}$, $\beta_{o}$, and $\delta_{o}$ have been linked to the input gate and are subject to training.

### 3.2 Convolutional neural network

CNNs are widely recognized as one of the primary types of neural network architectures employed for addressing various engineering tasks. The numerous advantageous characteristics exhibited by CNNs have contributed to their increased prominence in a wide range of computer vision applications. The network architecture is characterized by the inclusion of subsampling layers and convolution layers, which serve as fundamental components [48]. These layers are responsible for providing a detailed representation of the network structure. The LeNet-5 model is considered to be a straightforward yet highly efficient model within the recently introduced CNN models. The utilization of this network is attributed to its straightforward configuration, resulting in a reduced number of parameters for optimization [49].

The *k*th feature maps, illustrated by Eq. (7), are created by applying the hyperbolic tangent formula to the weights $w^{k}$ and biases *bk*. Furthermore, we can express the sub-sampling procedure as Eq. (8) [49].

(7) $$FM_{ij}^{k}=\tanh\;{(\left(W^{k}\times x\right)}_{ij}+b_{k})$$

(8) $$\alpha_{j}=\tanh\left(\beta \sum_{N\times N}\alpha_{i}^{n\times n}+b\right)$$

In the given equations, the variables *b* and β represent the bias values and learning variables, respectively, while the variable $\alpha_{i}^{n\times n}$ represents the inputs. In the end, the classification process is carried out by the highest-order fully-connected layer. The last layer's neuron count depends on the total number of output classes.

### 3.3 Slime mould optimizer

The fundamental concept underlying the SMO draws inspiration from the foraging behavior of slime mould in search of sustenance [21]. In instances where food sources are dispersed throughout an environment, the slime mould exhibits a mechanism by which it extends its network of protoplasmic tubes to establish connections between these food sources. Over a while, the network undergoes optimization processes to ensure the maintenance of the shortest routes to the most nutritionally rich food sources while simultaneously avoiding less lucrative areas. The equation governing the update process of slime moulds within the SMO is presented as Eq. (9) [21].

(9) $$\vec{X\left(t+1\right)}=\left\{ \begin{array}{c} \vec{X_{b}\left(t\right)}+\vec{vb}∙\left(\vec{W}∙\vec{X_{A}\left(t\right)}-\vec{X_{B}\left(t\right)}\right),r<p\\ \vec{vc}∙\vec{X\left(t\right)},r\geq p \end{array}\right.$$

In the given equation, the vector ($\vec{\;vb}$) represents a parameter that takes values within the interval [−a,a], while the vector ($\vec{vc}$) linearly decreases from a non-zero value to zero. Furthermore, let *t* represent the current iteration. The vector ($\vec{\;X_{b}}$) denotes the location of the individual currently identified as having the highest odor concentration. The vector ($\vec{X}$) represents the location of the slime mould. The vectors $\vec{X_{A}}$ and $\vec{X_{B}}$ represent two individuals randomly selected from the swarm. The weight of the slime mould is denoted as ($\vec{W}$), has been formulated in Ref. [21]. The variable p is formulated according to Eq. (10).

(10) p=tanh|S(i)−DF|

In this context, let *i* be an element belonging to the mentioned set. The fitness of $\vec{X}$ at iteration *i*, denoted by *S*(*i*), and the highest fitness achieved over all iterations, denoted by DF. In the equation mentioned above, the vector $\vec{vb}$ exhibits random oscillations within the interval [-*a*,*a*] and gradually converges to zero as the number of iterations increases. The magnitude of the vector ($\vec{vc}$) oscillates within the interval [-1,1] and ultimately approaches zero.

## 4 Proposed methodology

This section presents the evolving SMO with fuzzy systems and then evolving DLST_CNN using FUZ-SMO.

### 4.1 Evolving SMO with fuzzy systems

Each population member's current $\vec{vb}$ and $\vec{vc}$ values, as well as their Normalized Fitness Value (NFV), are factored into the fuzzy framework we suggest. The output specifies the size of the change using the symbols Δvb and Δvc. In addition, the NFV of each seeking agent can be determined with Eq. (11).

(11) $$NFV=\frac{fitness-{fitness}_{\min}}{{fitness}_{\min}-{fitness}_{\max}}$$

The value of NFV is within the range of 0.1. Eqs. (12), (13) denote the parameter update process for the vectors $\vec{vb}$ and $\vec{vc}$ in relation to each potential solution.

(12) $${\vec{vb}}^{t+1}={\vec{vb}}^{t}+\Delta vb$$

(13) $${\vec{vc}}^{t+1}={\vec{vc}}^{t}+\Delta vc$$

Initially, membership functions are employed to perform the process of *fuzzification* on these functions. The symbol *μ* afterward identifies the membership scores for these functions. According to a set of rules, these numbers are used to calculate Δvb and Δvc. Next, the defuzzification process is carried out to get a best guess about the values of Δvb and Δvc. Ultimately, the values are replaced in Eqs. (12), (13) to facilitate the update of Δvb and Δvc. The present study employs the Mamdani fuzzy mechanism.

**Fig. 3:**
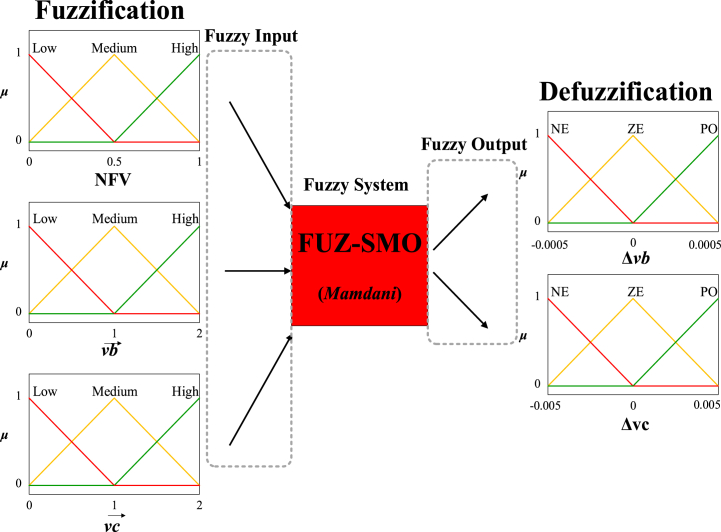
FUZ-SMO.

Low, Medium, and High are used as semantic values in the membership functions connected to Δvb and Δvc, as well as NFV. In addition, the semantic values NE (meaning Negative), PO (representing Positive), and ZE (representing Zero) are employed in the *vb* and *vc* output variables. The suggested fuzzy framework and membership functions for modifying the FUZ-SMO control parameters are depicted in Fig. 3.

### 4.2 Merging CNN with DLSTM

The suggested methodology involves the integration of CNN and DLSTM models. The fusion method encompasses the integration of CNN layers, which are purposefully designed to extract pertinent characteristics from the input data, with DLSTM models, which are primarily utilized for sequence forecasting tasks. As a result, the amalgamation of these elements yields a feature extraction technique that demonstrates a notable degree of efficacy.

The term “amalgamation” pertains to the fusion of CNN and DLSTM models, which have been specifically developed to process spectrograms as input data. The creation of the CNN-DLSTM model entails the incorporation of CNN layers in the early phase, followed by the integration of DLSTM layers and a dense layer at the end of the process. In this architectural configuration, two sub-models are utilized to obtain features and analyze them iteratively. The first sub-model, referred to as the CNN Model, is responsible for extracting features. The second sub-model, known as the DLSTM, is employed for the objective of feature analysis.

The model's architecture consists of a series of three consecutive layers of 2D convolution networks, which are subsequently accompanied by a pair of MaxPooling2D. The purpose of this arrangement is to attain the intended level of model depth. The layers mentioned above are tasked with examining the spectral attributes of the spectrograms, whereas the pooling layers facilitate the amalgamation of this study. The Conv2D layers are utilized to analyze the spectral characteristics of the spectrogram, while the pooling layers are applied to enhance and refine this analysis.

Initially, the input shape is processed by the Conv2D layer using 64 filters, a 5 × 5 kernel size, and one stride 1. Subsequently, a MaxPooling layer is employed to decrease the sizes of the input shape. The convolutional layer employs the ‘same padding’ approach to preserve the original height and width dimensions. The rationale behind selecting the ReLU activation function for this layer, instead of sigmoid units, is based on its intrinsic advantages, including improved efficiency in gradient creation and faster processing speed. Furthermore, in order to address the issue of overfitting, a rate of drop-out of 0.3 is implemented in the layers that utilize the same padding technique and ReLU activation function, similar to the inclusion of two extra Conv2D layers.

In order to initialize the DLSTM layers, a total of two levels are generated, with each layer including 128 hidden units. The ‘return sequence’ parameter is configured as true in order to provide the potential of stacking layers. In order to mitigate the issue of overfitting, a methodology is implemented wherein the outputs of the two DLSTM layers are converted into a three-dimensional tensor. The modified array is subsequently employed as the input for time-scattered dense layers, which have been designed with a drop-out rate of 0.2. The ReLU activation function is utilized in both deep DLSTM layers. The given input dimensions for the first layer of data are determined as 64 and 128, taking into account the input form of (21, 8).

The geometric configuration indicated above corresponds to a set of 20 iterations, providing the DLSTM model with essential information about the number of data points it must process when the input is applied. Following this, the output generated by the time-dispersed dense layer is passed into the flattened layer, and the iterative process persists until the desired outcome is achieved. Following the flattening procedure, the input data is converted into a vector format, which is then sent via the dense layer. The incorporation of the Softmax activation function in the dense layer enables the transformation of the given data into a discrete distribution of probabilities, which is then utilized as input for the dense layer.

In this work, the Adam optimizer is utilized as the selected optimization strategy owing to its capacity to assess the flexibility of parameters in response to variations in their surroundings. Adam demonstrates a superior level of competence compared to its predecessors since it presents a notably enhanced methodology for gradient descent. The methodology uses rates of adaptive learning to ascertain the optimal learning level for individual parameters. The research findings indicate that Adam demonstrates a predilection for error surfaces that possess flat minima, suggesting its proficiency in optimization.

The variables β_1_ and β_2_ have an indirect effect on the learning rate by determining the time that the learning rates decline. The correlation between rapid degradation and fluctuating learning rates, as well as the association between slow degradation and prolonged convergence of learning speed, has been established. The estimation of the learning rate is accomplished via an automated technique that depends on a dynamic computation of parameter gradients and squared gradients across different scenarios.

### 4.3 Problem definition

The input data for the CNN-DLSTM structure is expressed as a three-dimensional vector at each time step. This vector contains data pertaining to the number of time steps, the batch size, and the cells. During the training phase, it is conceivable that the DLSTM model might demonstrate a lack of responsiveness to the vector sound or have identified characteristics from the sonar audio dataset that are relatively consistent. The training procedure enables the DLSTM model to capture variations in sonar audio data successfully and construct complex regression associations by incorporating them into the model's output. The network topology of the DLSTM model is determined by hyperparameters, which exert a significant influence on the results of simulations. The batch size denotes the number of inputs that are simultaneously processed during training by the DLSTM structure. The time intervals govern the temporal resolution of data used for categorization. Within the specified temporal interval, the following values are dependent on the prior n values.

As the process of categorization progresses, the indicated values move down the time axis inside a window that moves of size *n* till they achieve the endpoint of the dataset. The presence of cells in the hidden layer of the DLSTM structure serves as an indication of the network's complexity and its capacity to acquire information. The hyperparameters have varied degrees of influence on the modeling outcomes for the task of sonar audio dataset categorization.

Sensitivity analysis is utilized to assess the influence of adjusting the hyperparameters on the results of the simulation. The word “lead time” is commonly used to refer to the elapsed duration between a projected point in time and the present instant. The assessment of sensitivity to simulation outcomes with different lead times is conducted by adjusting the numerical value of each hyperparameter within a predetermined range. In each iteration, a specific set of hyperparameters is chosen, and their values are adjusted within a predetermined range. The evaluation of the influence of hyperparameter adjustments on the results of the simulation is performed by employing the index of evaluation for the DLSTM-generated result as the target. This paper presents a novel methodology for classifying sonar data by integrating the FUZ-SMO technique with the DLSTM models.

This study aims to identify the optimal deep DLSTM network structure for categorization. Henceforth, the model under consideration is denoted as the CNN-DLSTM-FUZ-SMO framework. In simulation, the FUZ-SMO technique aims to optimize many parameters, including batch size, time steps, and cell number. The placement of each individual inside the algorithm is denoted by a three-dimensional variable, which corresponds to the time steps, the batch size, and the cell number in the DLSTM framework. The positional data of each individual is first set up at random within a predefined range of values.

**Fig. 4:**
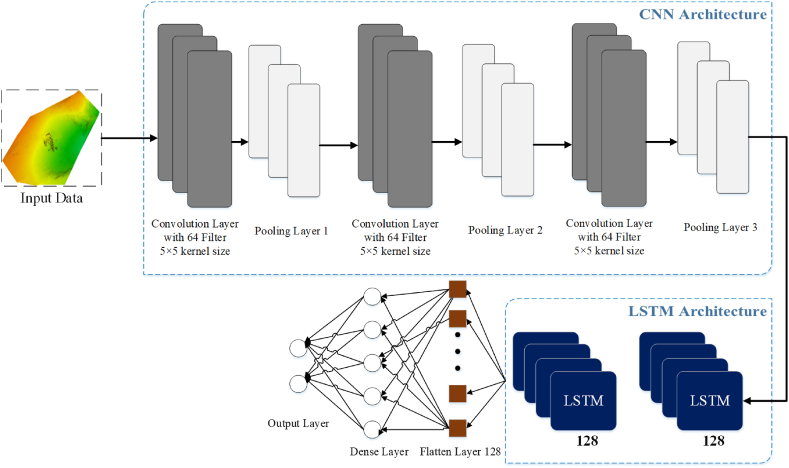
The proposed architectural layout.

The data that has been gathered is divided into two separate sets, namely the calibrating set and the validation set. The calibration set is utilized for training the CNN-DLSTM model, while the validation set is applied to evaluate the accuracy. The NSEF is employed as a measure to assess the fitness value of every individual's simulation results on the validation dataset. During every iteration, the location data of the individuals is consistently updated in order to determine the maximum fitness value. Fig. 4 illustrates the architectural layout of the proposed model. The pseudo-code of the proposed model is shown in Algorithm 1.

**Table:** 

Algorithm 1: Deep LSTM Hyperparameter Optimization
**Input**: *MaxIterations, FitnessFunction, HyperparameterRanges* **Output**: *OptimalHyperparameters* **Begin** **Initialize** Population with Hyperparameters within given ranges **Evaluate** Fitness of each Individual using *FitnessFunction* *BestAgentPosition* ← Find the best initial hyperparameters based on fitness **for***iter* = 1 to *MaxIterations* do **for** each Individual in Population do **Update** individual's position based on optimization strategy **Evaluate** Fitness of updated Individual **if** Fitness of updated Individual is better then **Update***BestAgentPosition* with updated individual **end if** **end for** **if** convergence criteria are met then break loop **end if** **end for** *OptimalHyperparameters* ← *BestAgentPosition* **Return***OptimalHyperparameters* **End**

### 4.4 Sonar datasets

This section represents the three sonar datasets, i.e., the Gorman & Sejnowski dataset, the Common dataset 2015 (CDS2015), and experimental datasets.

#### 4.4.1 Gorman & Sejnowski dataset

The initial data used in this study was obtained from the case study cited by Gorman and Sejnowski^1^ [50]. Two forms of backscatter are used in this experiment. A cylinder-shaped metal holds together the first type, whereas the second type is formed from a rock that is around the same size as the cylinder. First, on a sandy seafloor, we lay a metal cylinder measuring 5 feet in length and a rock of a comparable dimension as a control. Then, we send them a wideband FM chirp pulse (ka = 55/6).

**Fig. 5:**
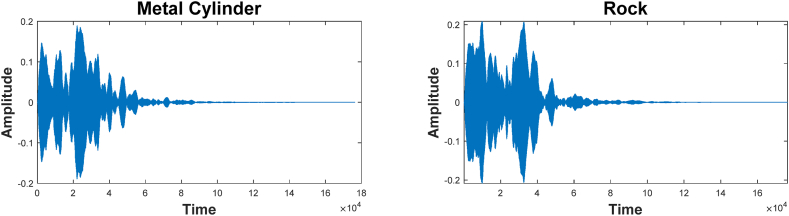
A sample of the backscatters received from the metal cylinder and the rock.

From the initially collected dataset comprising 1200 backscatter signals, a focused subset of 208 echoes was meticulously selected based on specific Signal-to-Noise Ratio (SNR) criteria, which were determined to range from 4 dB to 15 dB. This selection was guided by the objective of ensuring both a representative sample and high data quality. Within this refined subset, a balanced representation of the two categories was maintained: 111 echoes were identified as originating from the metal cylinder, and the remaining 97 echoes were attributed to the rock. This careful selection process aimed to provide a dataset that was not only representative of the diverse acoustic signatures of the two types of objects but also conducive to a robust and unbiased evaluation of our classification model. In Fig. 5, we see a representation of the backscatters obtained from the metal cylinder and the rock. Echoes from the cylinder and the solid material are practically identical, as seen in Fig. 5.

**Fig. 6:**
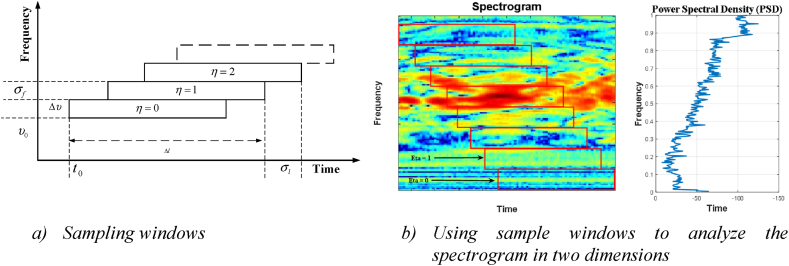
How a spectral envelope is prepared for use.

Preprocessing for getting the spectral envelope is shown in Fig. 6, a sampling window set is shown in Fig. 6a, and the sampling windows are shown on the sonar echo Fourier transform spectrogram in Fig. 6b. Thus, the spectrum is obtained by summing the results from all the windows. In this experiment, 60 spectral individuals with normalization factors between 0 and 1 are used to generate the spectral envelope. These values represent the total energy available during the sampling interval in question. For instance, the first of the 60 integers in the feature vector is the power of the first window (η=0) after normalization. References provide more information regarding the backscatters reflected from the cylinders and the rock.

#### 4.4.2 Common dataset 2015 (CDS2015)

The Common Dataset (CDS2015)^2^ [51] is the second dataset utilized to evaluate the custom-built CNN-DLSTM-FUZ-SMO. The purpose of this dataset is to make it easier to compare and contrast the various shallow water survey methodologies by providing a collection of echo datasets gathered using state-of-the-art techniques. Plymouth Sound and Wembury Bay were chosen because they offer a wide variety of depths, subsea Under certain circumstances, and seabed forms, making them ideal for this purpose. Shallow water is defined as water with a depth of less than 200 m. Nevertheless, in this set, the depths rarely exceed 40 m. Sub-40 m surveying has stringent criteria for navigational safety; therefore, only the highest quality data may be collected.

**Fig. 7:**
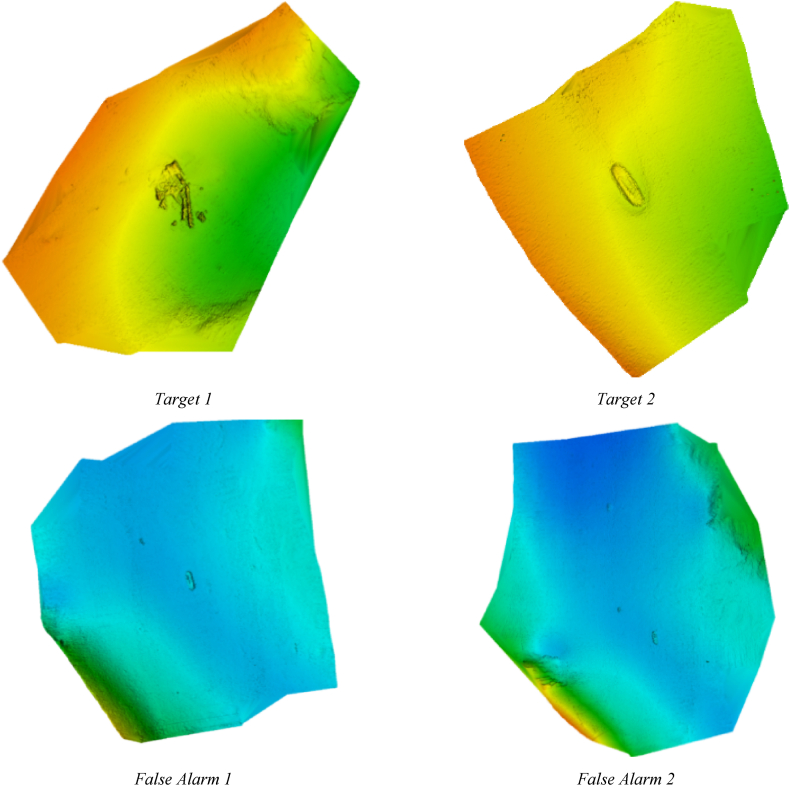
Four distinct goals for CDS2015.

Kongsberg Maritime's current technological abilities and advancements in low-depth water surveys are on display in this new dataset, which was made possible by their work in relatively shallow water. There have been no transformations or adjustments made to any of the offered data; they are all raw data. Therefore, CDS2015 is unprocessed data; no statistical or human filters have been applied. Data collection instructions show that all four targets were covered at the prescribed rate, with the prescribed swath angle, in the prescribed direction, and at the prescribed distance from the line. Additional information about the targets' descriptions can be found in the cited works. Fig. 7 displays the many target categories.

#### 4.4.3 Experimental underwater image dataset

**Fig. 8:**
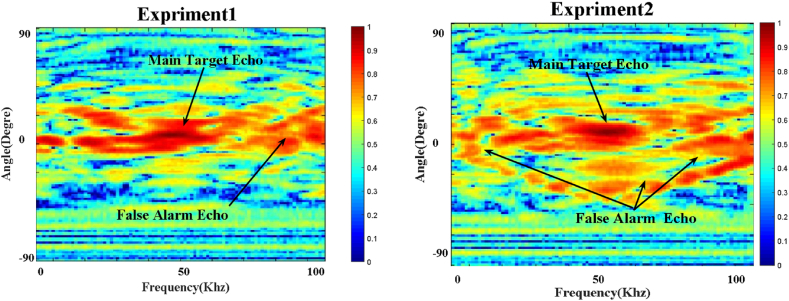
Comparison of target and non-target backscatters.

This brief section introduces the authors’ experimental dataset of underwater images.^3^ This data collection was compiled through field trials conducted near the coast of the Caspian Sea. The depth range here is only 40 m–100 m, making it a shallow water zone. Additional information on environmental factors such as seafloor type, velocity of wind, depth of water, salinity, and humidity has been collected from the oceanographic buoy operated by the Nowshahr Ports. Typical examples of target and non-target detected signals are shown in Fig. 8.

## 5 Experimentation and discussion

Python is used for model configuration and parameterization, and the Pandas, NumPy, and PySwarms modules are utilized for data preprocessing and management. Google TensorFlow, its deep learning platform, is used.

The values that can be used for the search parameters and the FUZ-SMO algorithm have been determined. A maximum of 200 iterations are allowed, and a population size of 30 is specified. Parameters like batch size [24, 128], number of cells [32, 256], and time steps [4,8] have defined optimization ranges based on characteristics of the time series data in the sonar audio dataset.

The developed models are used to optimize the model of sonar audio classifications. These models include CNN-DLSTM optimized by chimp optimization algorithm (CNN-DLSTM-CHOA) [52], slime mould optimizer (CNN-DLSTM-SMO) [21], arithmetic optimization algorithm (CNN-DLSTM-AOA) [53], prairie dog optimization algorithm (CNN-DLSTM-PDO) [54], marine predator algorithm (CNN-DLSTM-MPA) [55] and conventional CNN-DLSTM. The default parameters and fixed values of the categorization models are tabulated in Table 1.

**Table 1:** The setup parameters and initial values.

Algorithm	Parameter	Value
**MPA**	P EFIs	0.5 0.2
**FUZ-SMO**	z	0.1
**PDO**	Number of coteries specialized food source alarm stochastic property	30 100 Hz −1
**ChOA**	f μ	[2.5,0) Chaotic maps (Gauss/mauss)

In this study, several models were evaluated based on their RMSE, NSEF, and bias scores, all of which are statistical error metrics. Eqs. (14), (15), (16) are some definitions of these metrics:

(14) $$NSEF=1-\frac{\sum_{J=1}^{N}{\left(\xi_{0}-\xi_{c}\right)}^{2}}{\sum_{J=1}^{N}{\left(\xi_{0}-{\bar{\xi }}_{c}\right)}^{2}}$$

(15) $$RMSE=\sqrt{\sum_{J=1}^{N}\frac{{\left(\xi_{0}-\xi_{c}\right)}^{2}}{N}}$$

(16) $$bias=\frac{\sum_{J=1}^{N}\left(\xi_{0}-\xi_{c}\right)}{\sum_{J=1}^{N}\left(\xi_{0}\right)}$$

Where *N* represents the total number of data points, $\xi_{0}$ represents the actual values seen, ${\bar{\xi }}_{c}$ represents the average values observed, and $\xi_{c}$ represents the simulated values.

The NSEF measures the fraction of initial variation in a given variable that can be attributed to the model and provides an overall assessment of the model's predictive power. The closer a measurement is to 1 (perfect match), the more accurate the forecasts are expected to be.

The RMSE is a valid indicator of the accuracy of classification results since it is susceptible to extreme errors. If the RMSE numbers go down, then the classifications will get more precise.

The total accuracy of the water balance found in the simulation results is measured by the bias metric, which can take a value between −100% and 100%. The greater the precision of the classifications, the closer the value is to zero.

One, three, six, nine, and 12-h lead times were developed as comparison points against which the performance of several benchmarks could be assessed. Indicators of performance were computed and compared across all metrics in real-time.

### 5.1 The effect of hyperparameters

**Fig. 9:**
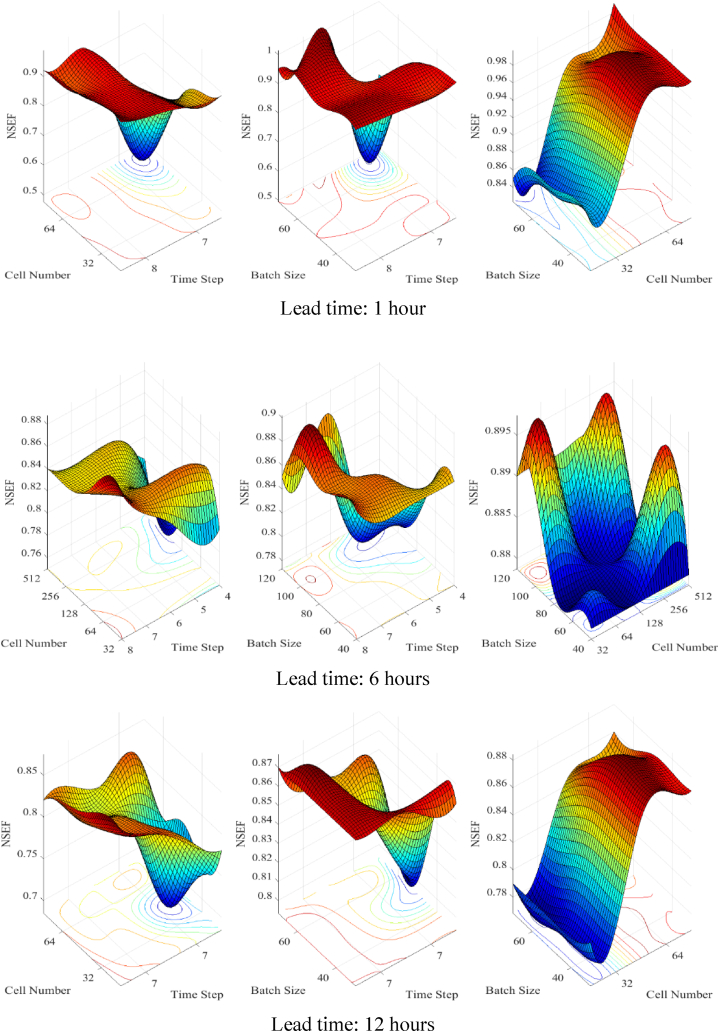
The effect of changing the hyperparameters on the results obtained with different lead times.

The hyperparameters were divided into three groups for analysis: time steps between 4 and 8, batch size between 24 and 128, and cell size between 32 and 256. Fig. 9 illustrates how changes to hyperparameters affect results for a range of lead times.

NSEF was shown to fluctuate between [0.9,1) depending on the hyperparameters with a 1-h head start. The results were susceptible to the cell count, with greater cell values resulting in higher and more stable NSEF once the cell count reached a particular threshold. However, when resources were limited, performance dropped significantly.

The effects of changing the time steps and batch size were not uniform, although they did seem to settle on a local maximum. The NSEF showed more variation when given a 6-h head start. While the effects of time step and batch size became more apparent, the cells consistently showed a positive connection with the NSEF.

With a day's notice, the NSEF's margin of error grew to [0.5, 0.75]. The hyperparameters had a major impact on the simulation results, and employing the wrong combinations affected the reliability of the simulation. The impact of hyperparameters on simulation results varied widely across different time frames. Better accuracy in short-term classification simulations was achieved by selecting appropriate hyperparameters. However, DLSTM networks have stricter requirements for greater lead times, which require the adoption of suitable hyperparameters.

### 5.2 CNN-DLSTM-FUZ-SMO parameter optimization

In this study, 30 searching agent served as independent variables, and their hyperparameters were each represented by three dimensions. The algorithm arbitrarily placed people in positions between zero and five. Using the NSEF of the sonar audio processing as the fitness value, the hyperparameters of the LSTM network were optimized for each classification time step.

**Fig. 10:**
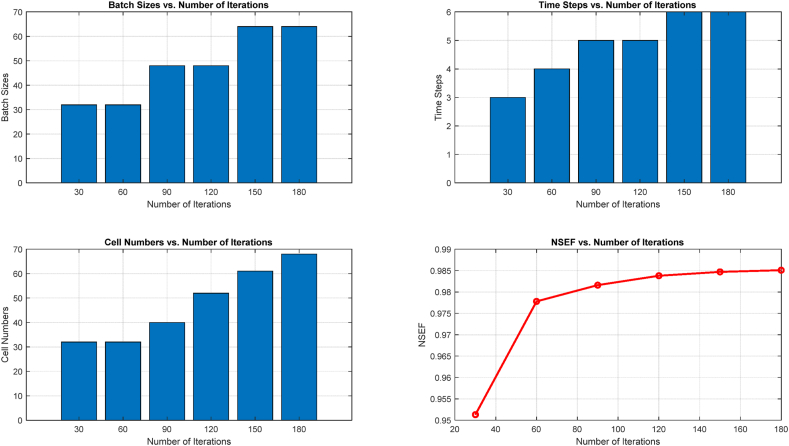
CNN-DLSTM-FUZ-SMO results.

The results of the CNN-DLSTM-FUZ-SMO approach are shown in Fig. 10 at various stages of the recursive procedure. As an example, we looked at how sonar acoustic processes are categorized 1 h into the future. The DLSTM model with a hidden layer was used, and 200 training iterations were performed. The hyperparameter settings of the DLSTM tended to converge on the ideal values as the number of iterations increased. The optimal set of hyperparameters for this task involved 68 neurons in the hidden layer, 64 samples in the batch, and six iterations. This particular combination, where the first six data points were used to predict the next data point, provided the highest accuracy in forecasting.

**Fig. 11:**
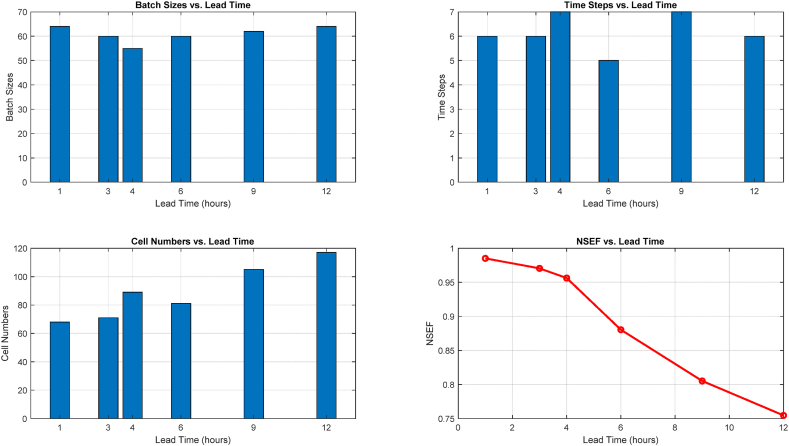
The outcomes of the hyperparameter optimization process for various lead times.

Hyperparameter optimization results were displayed in Fig. 11 and compared to a 1-h lead time for 1, 3, 6, 9, and 12-h lead times. When simulating the sonar sound process, the CNN-DLSTM-FUZ-SMO model performed quite well overall. There was an inverse relationship between the total time spent classifying and the quality of the results. After 1 h, the highest degree of classification accuracy was achieved, with an NSEF of 0.9877. The NSEF was 0.702 after 12 h.

The best batch size stayed close to 64 throughout all comparisons of best arrays of hyperparameters for different lead times. The optimal batch size was discovered to be dependent on data processing intensity in the DLSTM experiment. The computational complexity may rise if the batch size is too small and the DLSTM model cannot learn data patterns from a sufficient number of samples. For sonar audio categorization tasks, a batch size of 64 was shown to be optimal. The best classification accuracy was found for all lag times when there were roughly six steps in the experiment. The results show that the DLSTM model performed better when the input process classification considered a data sequence length of 6.

The optimal number of cells increased from 68 cells with 1 h of notice to 117 cells with 12 h of notice. The number of cells in a DLSTM network is indicative of its complexity and its ability to gather data characteristics. A lack of cells can provide a cognitive bottleneck in learning complex patterns, whereas an abundance of cells can cause overfitting. The model required a more significant number of cells as the lead time rose in order to get better predictions. The CNN-DLSTM-FUZ-SMO model performed well in predicting sonar audios, and the FUZ-SMO method was used to determine the optimal settings for the model's hyperparameters, resulting in improved accuracy in predictions.

### 5.3 Classification analysis

After sonar echoes have been preprocessed to achieve normalized data in the range (0, 1), the datasets from Gorman & Sejnowski (with dimensions of 208), CDS2015 (with dimensions of 625), and the generated dataset (with dimensions of 400) are applied to a CNN-DLSTM optimized with different methods. The outcomes are presented sequentially in Table 2, Table 3, Table 4.

**Table 2:** Experimental findings on the dataset created by Gorman and Sejnowski.

Model	MSE (AVE ± STD)	P-Values	Recognition Rate %
CNN-DLSTM-FUZ-SMA	1.10E-03 ± 0.0117	N/A	92.2100
**CNN-DLSTM-CHOA**	1.20E-03 ± 0.0251	0.021	90.4786
**CNN-DLSTM-AOA**	1.21E-03 ± 0.1014	0.042	89.3214
**CNN-DLSTM**	2.30E-03 ± 0.2745	0.0021	86.4241
**CNN-DLSTM-PDO**	1.22E-03 ± 0.0999	0.0011	89.9532
**CNN-DLSTM-MPA**	1.84E-03 ± 0.0998	0.0018	88.4138

**Table 3:** Observations on the CDS2015 dataset from experiments.

Model	MSE (AVE ± STD)	P-Values	Recognition Rate %
CNN-DLSTM-FUZ-SMA	1.04E-03 ± 0.0021	N/A	92.9985
**CNN-DLSTM-CHOA**	1.11E-03 ± 0.0101	0.0003	91.8585
**CNN-DLSTM-AOA**	1.12E-03 ± 0.1000	0.0021	90.3321
**CNN-DLSTM**	1.98E-03 ± 0.1546	0.41	88.2541
**CNN-DLSTM-PDO**	1.18E-03 ± 0.0903	0.21	89.3314
**CNN-DLSTM-MPA**	1.44E-03 ± 0.0925	0.0001	89.2241

**Table 4:** The created dataset's experimental results.

Model	MSE (AVE ± STD)	P-Values	Recognition Rate %
CNN-DLSTM-FUZ-SMA	1.02E-03 ± 0.0101	N/A	93.2144
**CNN-DLSTM-CHOA**	1.08E-03 ± 0.0251	0.41	91.1445
**CNN-DLSTM-AOA**	1.09E-03 ± 0.9874	0.47	90.3321
**CNN-DLSTM**	1.77E-03 ± 0.1332	0.0021	88.3322
**CNN-DLSTM-PDO**	1.22E-03 ± 0.0944	0.21	89.1111
**CNN-DLSTM-MPA**	1.35E-03 ± 0.1099	0.24	90.3352

Table 2, Table 3, Table 4 show experimental results on the Gorman and Sejnowski dataset, the CDS2015 dataset, and the newly constructed dataset, respectively, demonstrating the efficacy of the proposed CNN-DLSTM-FUZ-SMA model compared to other versions. We go over the main points and what they mean in this part.Our CNN-DLSTM-FUZ-SMA model outperformed other models in terms of prediction accuracy in the experiments conducted on the Gorman and Sejnowski dataset, with an MSE of 1.10E-03 ± 0.0117. The recognition rate of 92.21% supports the model's resilience in sonar sound dataset classification. Because of its novel optimization strategy, the CNN-DLSTM-FUZ-SMA model does not present itself to statistical analysis based on P-values.

Other models, such as CNN-DLSTM-CHOA and CNN-DLSTM-AOA, had slightly higher MSE values and achieved lower recognition rates during comparison. According to the P-values, CNN-DLSTM-FUZ-SMA is significantly better than these options.

Results on the CDS2015 dataset show that CNN-DLSTM-FUZ-SMA consistently outperforms its competitors. The model's ability to handle varied datasets is demonstrated by its identification rate of 92.99% and MSE of 1.04E-03 ± 0.0021.

Other models, on the other hand, have lower recognition rates and larger MSE values. Notably, CNN-DLSTM-AOA and CNN-DLSTM-PDO show more variability, which indicates that they are more affected by the peculiarities of the dataset. The P-values for various models are statistically significant, which further supports the idea that they function differently.

The newly generated dataset further proves that CNN-DLSTM-FUZ-SMA is a robust model. The model consistently achieves better results than other variations, with an MSE of 1.02E-03 ± 0.0101 and a recognition rate of 93.21%. The fact that the model achieves excellent accuracy. Although CNN-DLSTM-CHOA and CNN-DLSTM-AOA are models that show competitive performance, the statistical analysis brings more subtlety to their relative efficacy. It is worth mentioning that CNN-DLSTM-CHOA outperforms CNN-DLSTM-AOA statistically, which emphasizes the significance of model selection in some situations.

The fact that CNN-DLSTM-FUZ-SMA consistently outperforms other models on different datasets indicates that it is versatile and successful for sonar sound classification tasks. Relying less on statistical comparisons, the stability and dependability of this model are indicated by the absence of P-values. To conclude, the CNN-DLSTM-FUZ-SMA model is an effective and promising way to improve the accuracy and efficiency of sonar sound dataset classification. Faster convergence, resistance to overfitting, and enhanced classification accuracy are all outcomes of integrating long-short-term memory and convolutional networks with the novel FUZ-SMA optimization strategy. The transferability of this approach to different fields and data sets might be the subject of future studies.

**Fig. 12:**
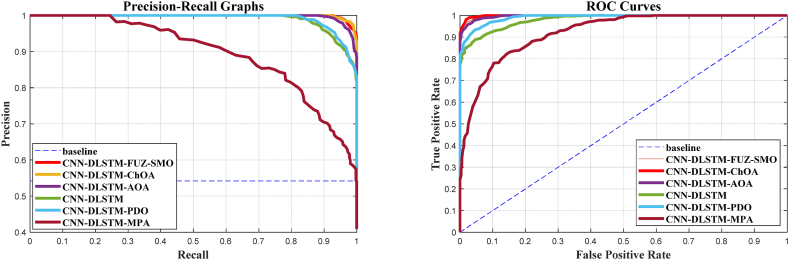
Gorman & Sejnowski dataset.

**Fig. 13:**
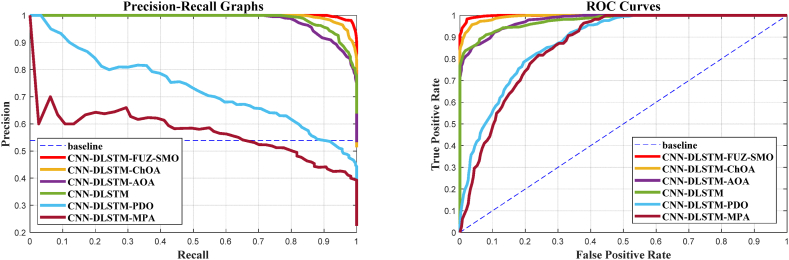
CDS2015 dataset.

**Fig. 14:**
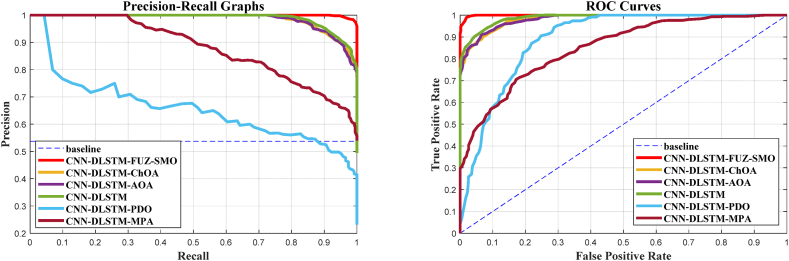
Developed dataset.

It has been claimed that a highly efficient exploration phase approach is required for sonar classification since it spans the whole search space; FUZ-SMO demonstrates outcomes that are relatively equivalent to or beyond those of other techniques. It is not adequate to compare algorithms based only on their recognition rates because the detection performance of the detection system is dependent on the threshold level. When evaluating these systems over all possible threshold options, the precision-recall graph is a good indicator. The accuracy-recall curves for the six standard models are shown in Fig. 12, Fig. 13, Fig. 14. The ROC curve is another helpful way to visualize the TPR in relation to the FPR. In Fig. 12, Fig. 13, Fig. 14, we see the ROC curves for the reference models and FUZ-SMO.

The ROC curves for three different sonar test datasets show that the FUZ-SMO technique outperforms both the reference methods and classical SMO. However, it should be noted that the Area Under Curve (AUC) associated with the ROC plots may not adequately indicate the efficacy of the model for severely imbalanced test sets.

The following sentences have been incorporated to highlight the key strengths of the study explicitly:

**Robust Methodology:** This study utilizes a strong and established approach that combines CNN and LSTM networks with a unique FUZ-SMA optimization strategy. By combining these components, a thorough method for classifying sonar sound datasets is established, improving the model's flexibility and forecasting precision.

**Novel FUZ-SMA Optimization Strategy:** The proposed approach's intrinsic strength is the innovative FUZ-SMA optimization mechanism it utilizes. This strategy shows outstanding efficiency in thoroughly investigating the whole search space, resulting in outcomes that are equal to or better than those of other well-known methods. FUZ-SMA's versatility across various datasets and its capacity to match or exceed current methods establish it as a significant addition to the field.

**Comprehensive Comparative Analysis:** A comprehensive comparative analysis was undertaken, wherein the CNN-DLSTM-FUZ-SMA model was evaluated in relation to several cutting-edge alternatives. The thorough evaluation involves statistical measures, recognition rates, and MSE on various datasets such as Gorman and Sejnowski, CDS2015, and a freshly created dataset. The proposed approach consistently outperforms others in classifying sonar sound datasets, demonstrating its superiority.

**Statistical Significance and Rigorous Evaluation:** The results are supported by thorough statistical analyses, which include p-values and confidence ranges, to ensure the strength and statistical significance of our findings. Integrating statistical metrics enhances the credibility of our methodology and establishes a strong foundation for the validity of our proposed strategy.

**Transferability Across Datasets:** The proposed methodology has shown success across a variety of datasets, including Gorman and Sejnowski, CDS2015, and a newly created dataset. The reliable performance of CNN-DLSTM-FUZ-SMA highlights its adaptability and suitability for different sonar sound categorization tasks, positioning it as a potential solution for practical use.

**Improved Accuracy and Efficiency:** The CNN-DLSTM-FUZ-SMA model typically outperforms other versions in terms of accuracy and efficiency, as demonstrated by recognition rates and MSE values. The model's resistance to overfitting, faster convergence, and improved accuracy makes it successful in handling the intricacies of sonar sound datasets.

Overall, these emphasized strengths establish this study as an essential addition to the field of sonar sound dataset classification. The integration of a strong methodology, an innovative optimization strategy, and a thorough comparison study support the effectiveness and relevance of the suggested CNN-DLSTM-FUZ-SMA model.

• **Addressing Limitations**

The paper acknowledges and discusses the limits of the technique to ensure transparency and a thorough understanding. **Generalization to Other Domains**: Although the CNN-DLSTM-FUZ-SMA model has shown effectiveness in classifying sonar sound datasets, its ability to perform well in different domains could be influenced by differences in data features. The distinctive attributes of sonar audio recordings, like temporal patterns and signal characteristics, could hinder the direct applicability of the method to various datasets. **Hyperparameter sensitivity:** Hyperparameter sensitivity is crucial for optimizing the model's performance. Even though a thorough examination of hyperparameter impacts has been carried out, differences in dataset attributes may require particular modifications. The sensitivity to hyperparameters highlights the need of careful parameter tweaking for best results.

**Computational Complexity:** The high computational demand of the method, especially during the optimization phase, could be challenging to manage in contexts with limited resources. Convergence time and resource demands should be taken into account in practical applications with potential constraints on processing resources.

**Lack of Real-Time Considerations:** The study mainly concentrates on analyzing previous data and performing classification tasks without taking real-time factors into account. The model's suitability for real-time situations and dynamic contexts has not been thoroughly investigated. Challenges related to real-time implementation should be further investigated.

**Dataset Dependency:** The model's success relies on the specific attributes of the datasets utilized for training and assessment. Extending the results to datasets with notably different characteristics may necessitate further verification and adjustment of the model structure.

These limitations are essential factors to take into account when interpreting and applying the methodology. The purpose is to recognize these limitations in order to steer future research paths and promote a detailed comprehension of the extent and restrictions of the suggested strategy.

## 6 Conclusion

The investigation of sonar sound dataset classification via the combination of Long-Short Term Memory (LSTM) and Deep Convolutional Neural Networks (CNNs), along with the creative use of the Fuzzy Slime Mould Optimizer (FUZ-SMO) for LSTM hyperparameter tuning, opens up new avenues for the refinement and enhancement of sound classification methods in submerged environments. This novel method makes extensive use of slime moulds’ adaptive behavior, converting their innate tendencies into a stable and dynamic optimization model. The resulting combined model delivers a remarkable 2.142% improvement in classification accuracy over conventional LSTM frameworks. Furthermore, the model not only achieves faster convergence milestones but also strengthens against common overfitting behaviors, proving to be a dependable and effective technique for sonar sound classification.

Even though the results are encouraging, future research into improving the FUZ-SMO may reveal other possibilities and lead to even more accurate LSTM hyperparameter tuning. Enhancing adaptive reactions could entail looking into various fuzzy logic systems or further simulating biological slime mould behaviors.

If different neural network designs or machine learning models are added, the combined model may be appropriate for different applications in marine research and other fields where sophisticated sound dataset classification is needed.

Research might examine how well the model performs in a range of undersea settings and environments by examining its adaptability and application across different kinds and quality of sonar sound datasets.

Clarifying the combined model's decision-making process is essential for using it in scientific and surveillance settings. Enhancing the model's interpretability and explainability should be the top priority for future study. This interpretability and explainability might be done by exploring different, more interpretable model topologies or by incorporating visualization approaches.

In particular, in surveillance and urgent research contexts, investigating the model's feasibility in real-time sonar sound classification and putting it into edge computing scenarios for on-site, instant analysis in underwater operations could prove invaluable.

Preserving the integrity and reliability of the model during real-world deployment will require examining and strengthening it against adversarial attacks as well as guaranteeing its resilience, particularly in dynamic and possibly unstable underwater conditions.

One potential limitation of our study is that DCNNs and LSTM models require a large quantity of training data in order to utilize their complex structure and avoid overfitting effectively. Although our datasets are robust and have been proven sufficient for the extent of our research, there is still a possibility of ‘attention drift’ or disappearing gradients, especially when using LSTM models since they handle temporal data. We have employed various well-established strategies to alleviate these dangers; nonetheless, it is essential to note that these procedures are not a universal solution. Potential future research might investigate the utilization of attention processes and more sophisticated regularization approaches to further improve the model's capacity to learn from intricate data without succumbing to overfitting, mainly when dealing with larger datasets.

Future research can significantly improve and modify the current model by following these suggested directions, ensuring that it continues to be technologically innovative and environmentally responsible while also broadening its scope of application across a variety of real-world uses and avenues for scientific inquiry in marine and underwater environments.

The suggested methodology, which combines DLSTM with CNN for the classification of sonar sound datasets, exhibits considerable promise in practical underwater applications. The utilization of this technology in edge computing situations offers significant benefits, especially for conducting real-time analysis on-site in underwater operations, including naval surveillance, environmental monitoring, and marine life research. Deploying this model on edge devices would facilitate swift, on-site analysis of sonar data, diminishing delays and dependence on cloud-based systems.

## Funding

This work was supported by Key Scientific Research Projects of Colleges and Universities of Henan Province: Application research of multi-view feature dimensionality reduction technology in image classification (No. 22A520052).

## Data availability statement

The datasets presented in this article are publicly available by the following links:

https://archive.ics.uci.edu/dataset/151/connectionist+bench+sonar+mines+vs+rocks

https://www.hydro-international.com/content/news/shallow-survey-2015-common-dataset-collection-has-begun

https://data.mendeley.com/datasets/fyxjjwzphf/1.

## Ethics statement

Ethics Statement: This research did not involve any human participants or animals, and as such, ethical approval was not applicable to this study.

## Additional information

No additional information is available for this paper.

## CRediT authorship contribution statement

**Dong liang Zhang:** Formal analysis, Data curation. **Zhiyong Jiang:** Resources, Methodology, Investigation. **Fallah Mohammadzadeh:** Project administration, Conceptualization. **Seyed Majid Hassani Azhdari:** Validation, Software, Methodology. **Laith Abualigah:** Writing – review & editing, Visualization, Validation, Conceptualization. **Taher M. Ghazal:** Writing – review & editing, Validation.

## Declaration of competing interest

The authors declare that they have no known competing financial interests or personal relationships that could have appeared to influence the work reported in this paper.

## Footnotes

1 The dataset is available for download at the following link: https://archive.ics.uci.edu/dataset/151/connectionist+bench+sonar+mines+vs+rocks.

2 The dataset can be found at the following link: https://www.hydro-international.com/content/news/shallow-survey-2015-common-dataset-collection-has-begun.

3 The dataset is available for download at the following link: https://data.mendeley.com/datasets/fyxjjwzphf/1.
